# Identification of a TRP channel-related risk model for predicting prognosis and therapeutic effects of patients with hepatocellular carcinoma

**DOI:** 10.1007/s00432-023-05394-7

**Published:** 2023-09-21

**Authors:** Chong Pang, Zhe Xu, Jilong Han, Fujun Li, Hongyan Zhu, Jiaqi Zhang, Dong Wang, Xundi Xu

**Affiliations:** 1 Department of Hepatobiliary Pancreatic Surgery, South China Hospital, Health Science Center, Shenzhen University, Shenzhen, 518116 People’s Republic of China; 2 Department of Breast and Thyroid Surgery, South China Hospital, Health Science Center, Shenzhen University, Shenzhen, 518116 People’s Republic of China

**Keywords:** Transient receptor potential, Hepatocellular carcinoma, Therapeutic effects, Prognosis prediction, Bioinformatic algorithm

## Abstract

**Purpose:**

TRP channels have been implicated in cancer progression. Our study seeks to establish a prognostic model for hepatocellular carcinoma (HCC) by utilizing genes related to TRP channels.

**Methods:**

We used the TCGA and ICGC databases as training and validation cohorts, respectively. We calculated the risk scores using Lasso–Cox regression analysis based on the expression levels of prognostic genes and performed survival analysis to compare overall survival between high- and low-risk groups. Then we compared the clinicopathologic characteristics and conducted biological functional analysis. We also explored immune cell infiltration and compared the drug sensitivity.

**Results:**

Using bioinformatics algorithms, we identified 11 TRP-related genes and calculated the risk scores. Patients in the high-risk group demonstrated worse overall survival, as well as more advanced T stage and pathologic stage. The risk score showed a significant association with the cell cycle. The high-risk group had more ICI and RTK targets with elevated expression and showed better therapeutic effect to chemotherapy including 5-fluorouracil, camptothecin, docetaxel, doxorubicin, gemcitabine, and paclitaxel. Overall, an individualized nomogram was constructed by integrating the risk score and requisite clinicopathologic parameters to predict the overall survival of HCC patients.

**Conclusions:**

We successfully established a highly accurate prognostic model for predicting overall survival and therapeutic effects using TRP channel-related genes.

**Supplementary Information:**

The online version contains supplementary material available at 10.1007/s00432-023-05394-7.

## Introduction

Primary liver cancer is globally recognized as the sixth most common cancer type and ranks as the third most common cause of cancer-related mortality (Sung et al. 2021). Hepatocellular carcinoma (HCC) is the most prevalent primary liver cancer and typically arises in the context of chronic liver disease caused by hepatitis B or C virus infection, alcohol abuse, or metabolic syndrome (Llovet et al. 2016). Although the mortality rate of liver cancer has been increasing for decades, the rates have stabilized during the most recent 5 years in both men and women owing to advances in early detection, surgical techniques, and molecularly targeted therapies (Siegel et al. 2022). Despite advancements in treatment options, the overall survival rate of HCC patients remains unsatisfactory due to recurrent disease, metastasis, and drug resistance. The discovery of new targets is needed to slow down disease progression and improve prognosis.

Transient receptor potential (TRP) channels were initially discovered in a blind strain of *Drosophila* (Montell and Rubin 1989) and are now recognized as a family of ion channels with versatile functions. Structurally, TRPs are characterized by six transmembrane spanning domains (S1–S6) and are classified into eight families: TRPA (ankyrin), TRPC (canonical), TRPM (melastatin), TRPML (mucolipin), TRPN (NO-mechano-potential), TRPP (polycystin), TRPS (soromelastatin), and TRPV (vanilloid) (Wu et al. 2010; Zhang et al. 2022). Functionally, TRPs are gated by various stimuli, including thermal, pain, mechanical, and chemical inputs and function as intracellular ion channels in cellular organelles such as lysosomes, the Golgi network, and the endoplasmic reticulum (Gees et al. 2010; Himmel and Cox 2020).

During tumor formation and metastasis, abnormal TRP expression has been observed in multiple cancers, which may act as a vital role of promoting the proliferation and metastasis of cancer (Brooks et al. 2010; Chen et al. 2014). We previously demonstrated that TRPV2 knockdown enhances the stemness of cancer stem-like cells through increased expression levels of cancer stem cell markers ALDH1, CD133, and CD44 (Hu et al. 2018). Additionally, the overexpression of TRPV2 attenuated the stemness of cancer stem-like cells by reducing marker levels. Koh et al. revealed that low TRPV6 expression was remarkably lined to adverse histologic features, resulting in the worse prognosis (Koh et al. 2022). Similarly, Xu et al. showcased that TRPC6 and the Na + /Ca2 + exchanger 1 (NCX1) mediated the effects of TGF-β on the migration, invasion, and intrahepatic metastasis of human HCC cells (Xu et al. 2018). However, the number of experimental studies on TRPs and HCC remains limited. The biological functions of most members of TRPs and TRP-related genes during the development of HCC require more explorations.

Building upon previous research, we managed to elucidate the therapeutic and prognostic effects of TRP-related genes (TRGs) on HCC. To achieve this, we collected and analyzed transcriptome profiling data from HCC patients obtained from The Cancer Genome Atlas (TCGA) and the International Cancer Genome Consortium (ICGC). Based on this data, we intended to construct a prognosis model that incorporates TRGs and clinicopathologic factors. Our model exhibits high accuracy to forecast the survival status of HCC patients. Overall, our findings suggest that TRGs have a substantial impact on the choice of therapy and clinical outcome for patients with HCC, consequently providing new insights into the management of this disease.

## Materials and methods

### Acquisition of datasets

120 TRGs were acquired from two gene sets: “Reactome_TRP_channels” in the molecular signatures database (MSigDB) and “inflammatory mediator regulation of TRP channels” in the Kyoto Encyclopedia of Genes and Genomes (KEGG) database (Supplementary Material 1). Gene expression data and clinical information of patients with HCC were obtained from TCGA-LIHC, and somatic mutation data were acquired from the UCSC Xena repository. After eliminating patients with missing survival information and overall survival of 0, we collected 354 HCC and 49 normal cases with relevant clinicopathologic information and gene expression profiles (HTSeq-FPKM) as well as 364 HCC cases with somatic mutation for further analysis as the training cohort. The corresponding data downloaded from ICGC-LIRI were used as the validation cohort.

### Biological functional analysis

Gene ontology (GO) and KEGG pathway analyses were applied to examine the functions of 120 TRGs, using the R package “clusterProfiler” and Database for Annotation, Visualization and Integrated Discovery.

### Calculation of TRG-based risk score for HCC

We conducted the least absolute shrinkage and selection operator (LASSO) Cox regression analysis on the training cohort to minimize redundant TRGs using the R package “glmnet.” A prognostic model was fitted using the Cox proportional hazards model, based on the TRGs expression and survival information of each individual. The fitting process was performed with a maximum of 1000 iterations. The value of *λ* corresponding to the minimum penalized likelihood deviation was chosen as the optimal *λ*. We identified 11 TRGs for the establishment of the prognosis model. The risk score for each patient was calculated based on the expression of TRGs and their corresponding regression coefficients. The calculation formula was as follows:

$$\mathrm{Risk} \,\mathrm{score}=\sum_{\delta =1}^{11}\left(\mathrm{Exp}\delta *\mathrm{Coe}\delta \right).$$

Exp*δ* indicates the expression of each TRG, and Coe*δ* represents the corresponding Cox regression coefficient.

Subsequently, patients in the training cohort were divided into high- and low-risk groups based on the median risk score. The validation cohort was processed similarly. R package “survminer” and “timeROC” were applied to conduct the survival analysis and time-dependent receiver operating characteristic (ROC) curve analysis, respectively. The difference of prognosis was analyzed, and the performance of the prognostic models was assessed for predicting survival outcomes at different time points.

### Immune infiltration analysis

To calculate the proportion and expression of immune cells infiltrating HCC tumors for each patient, we utilized two methods: cell-type identification by estimating relative subsets of RNA transcripts (CIBERSORT) and single-sample gene set enrichment analysis (ssGSEA) (Bindea et al. 2013; Hänzelmann et al. 2013; Newman et al. 2015). We then performed the correlation analysis between 11 TRGs and immune cells using Spearman’s coefficient and visualized the correlation.

### Tumor mutation burden (TMB) analysis

After removing samples with incomplete data, we utilized the R package “maftools” to analyze simple nucleotide variation data (VarScan2) from 343 patients with LIHC. To explore differences in tumor mutation burden (TMB) between the two groups, we visualized the top 20 genes with the highest mutation rates using separate waterfall plots.

### Differentially expressed genes and GSEA

We conducted differential analysis using the R package “limma” on the expression profiling data (HTSeq-FPKM), setting the cutoff value at *p < *0.05. To identify biological processes and pathways enriched in the two groups, we used gene set enrichment analysis (GSEA). We considered gene sets with a nominal |NES|> 1 and *p < *0.05 to represent significant enrichment of the biological process.

### Analysis of drug sensitivity

We utilized the half-maximal inhibitory concentration (IC50) data from the Genomics of Drug Sensitivity in Cancer (GDSC) database to compare the chemosensitivity between the two groups.

### Construction of the prognostic model

A nomogram was established using R package “rms.” The total score of each prognostic factor in the scoring system corresponded to the estimated 1-, 2-, 3-, and 5-year survival of HCC patients at in the prediction system. Decision curve analysis and calibration curves were used to indicate the accuracy of the survival prediction.

### Statistical analysis

Statistical analyses were conducted using the R (4.2.1) software. Figures were created using Photoshop (2019). Mann–Whitney test was used to indicate the significance of the difference between the two groups, whereas that between three or more groups was verified using the Kruskal–Wallis test. Spearman’s coefficient was applied to perform correlation analysis. The clinicopathologic features between the two groups were compared using the Chi-square test, and Fisher’s exact test was applied when required. All hypothetical tests were two-sided, and *p < *0.05 was considered to indicate statistically significant differences.

## Results

### Identification of TRGs

**Fig. 1:**
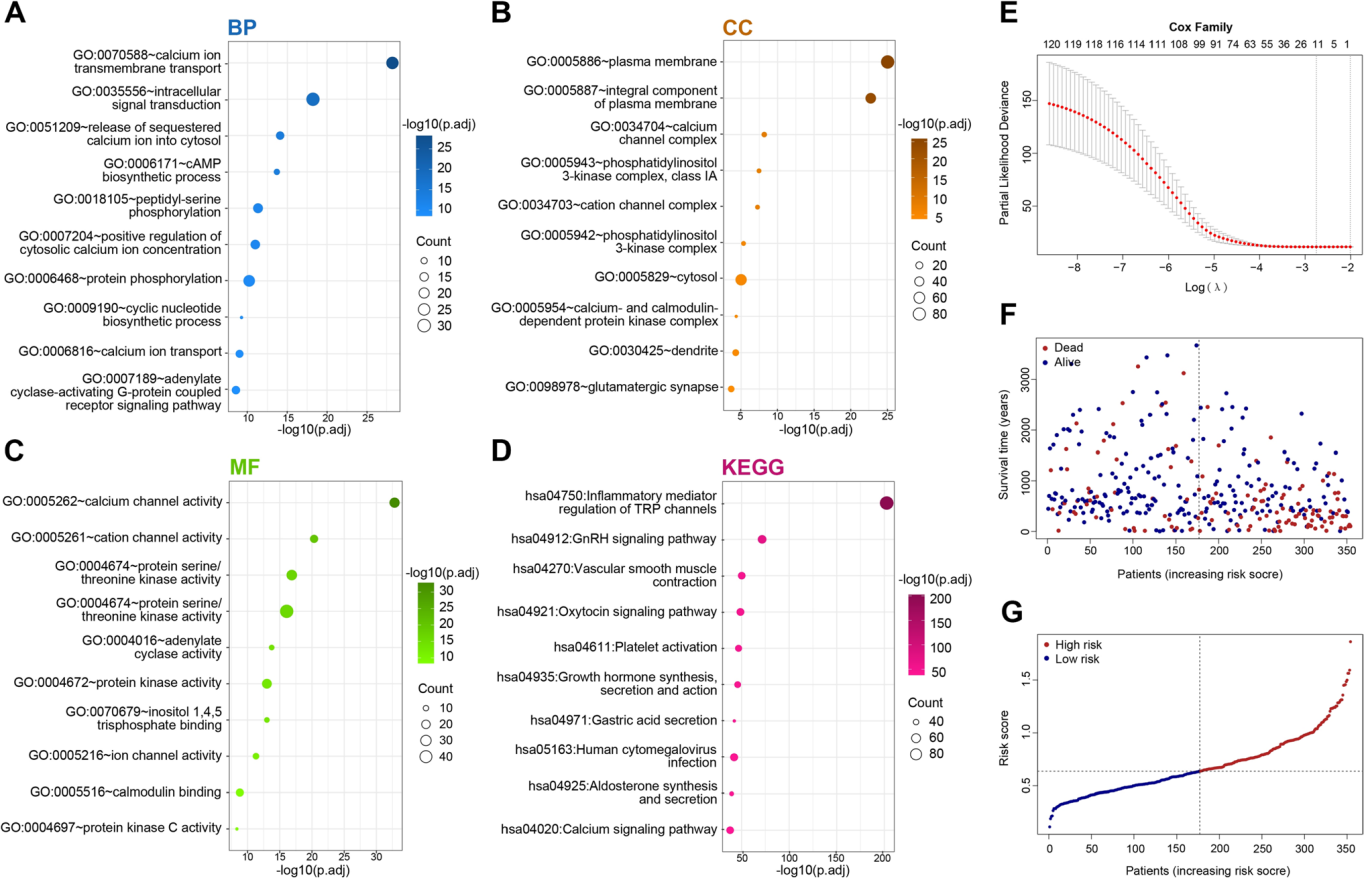
Biological functional analysis of 120 TRP and identification of TRGs. **A–D** GO and KEGG analysis of 120 TRP. **E–G** Lasso–Cox regression analysis of TRGs

We comprehensively explored the biological processes and signaling pathways associated with 120 TRGs by conducting GO and KEGG analyses. The most significant GO terms were calcium channel activity, calcium ion transmembrane transport, protein serine/threonine kinase activity, and intracellular signal transduction (Fig. 1A–C). The KEGG analysis revealed that several signaling pathways, including inflammatory mediator regulation of TRP channels, calcium signaling pathway, and vascular smooth muscle contraction, were enriched (Fig. 1D). Subsequently, the following 11 TRGs were identified via LASSO regression analysis: *PIK3R1, PLCB1, PLCB3, PPP1CB, PPP1CC, PRKCD, PRKCQ, RIPK3, TRPC4AP, TRPM1,* and *TRPM6* (Fig. 1E). We figured out the risk score based upon the expression of 11 TRGs and their regression coefficients:

$$\begin{gathered} {\text{risk}}\,{\rm{ score }} = ( - \,0.013979829 \times _{{{\text{Exp}}}} PIK3R1) + (0.026509423 \times _{{{\text{Exp}}}} PLCB1) + (0.004029417 \times _{{{\text{Exp}}}} PLCB3) + (0.013928081 \times _{{{\text{Exp}}}} PPP1CB) + (0.016535821 \times _{{{\text{Exp}}}} PPP1CC) + (0.006513565 \times _{{{\text{Exp}}}} PRKCD) \hfill \\ + ( - 0.032692058 \times _{{{\text{Exp}}}} PRKCQ) + ( - 0.058352729 \times _{{{\text{Exp}}}} RIPK3) + (0.010791336 \times _{{Exp}} TRPC4AP) + ( - 0.323784473 \times _{{{\text{Exp}}}} TRPM1) + (0.037941235 \times _{{{\text{Exp}}}} TRPM6). \hfill \\ \end{gathered}$$

**Fig. 2:**
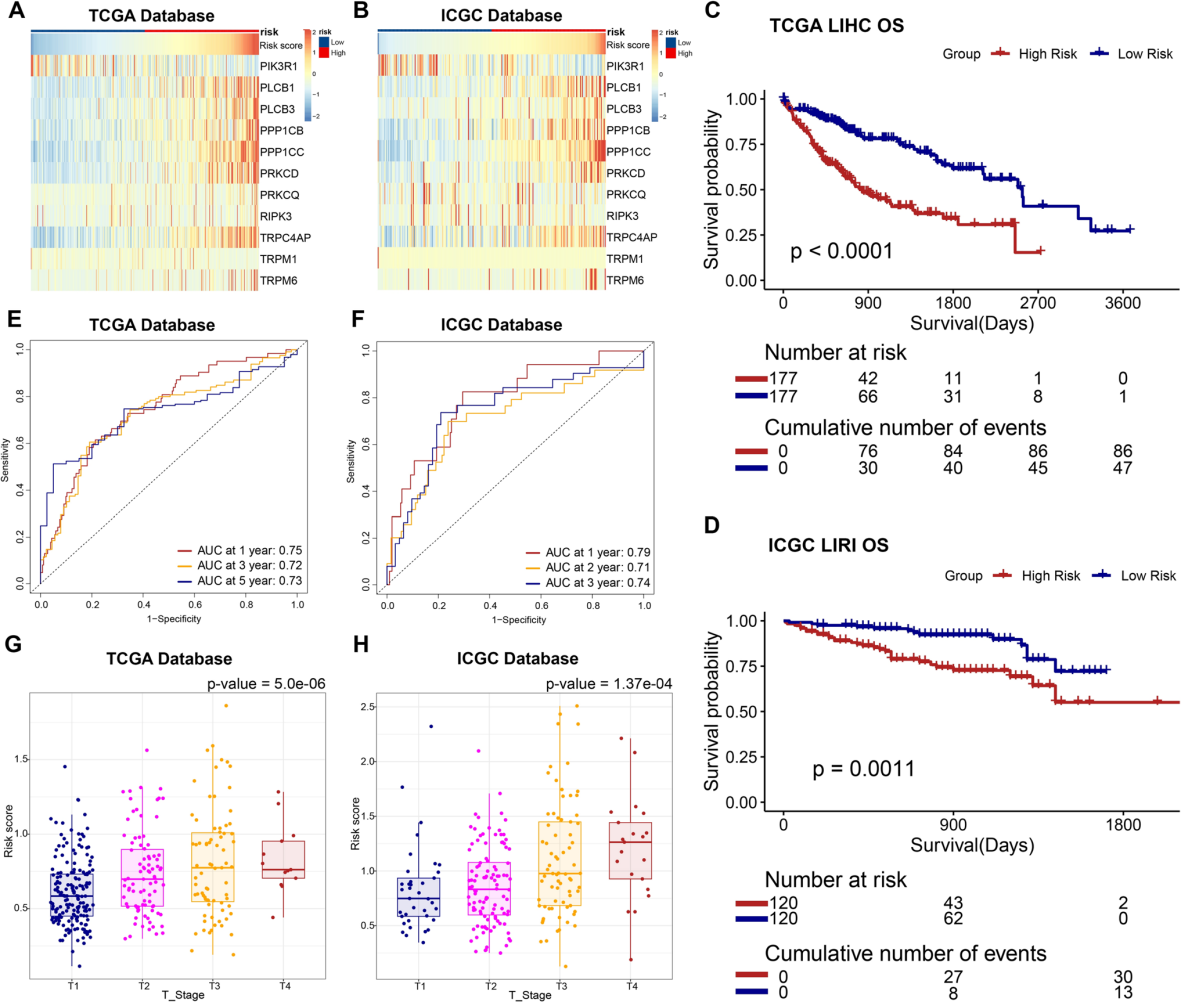
Comparison of clinicopathologic characteristics between the two groups. **A**, **B** Expression landscape of 11 TRGs. **C**, **D** Survival analysis of TCGA–LIHC and ICGC–LIRI patients. **E**, **F** Time–ROC analysis. **G**, **H** Comparison of risk score in different T stages

The risk score was used to divide the training and validation groups into high- and low-risk groups, respectively. Figure 1F, G displays the survival information and risk scores for each sample. Heatmaps (Fig. 2A, B) shows the expression of the 11 TRGs between these two risk groups.

### Correlation between risk score and clinicopathologic characteristics

We compared the clinicopathologic characteristics of the two groups based on the risk scores of the TCGA–LIHC samples. The ICGC–LIRI samples were processed similarly. Our analysis revealed that compared with the low-risk group, the high-risk group had significantly worse overall survival (Fig. 2C, D). Furthermore, the time–ROC results yielded satisfactory predictive accuracy of the risk score for overall survival (Fig. 2E, F). Patients in the high-risk group exhibited higher pathological T stage (Fig. 2G, H). However, no significant differences were observed with regard to gender, N/M stages, treatment, or age between the two groups (Table 1, Table S1).

**Table 1:** Clinicopathologic characteristics of the two groups

	ALL	Low risk	High risk	*p*. overall
	*N* = 354	*N* = 177	*N* = 177	
OS Time	820 (731)	983 (819)	657 (591)	< 0.001
OS				< 0.001
Alive	226 (63.8%)	132 (74.6%)	94 (53.1%)	
Dead	128 (36.2%)	45 (25.4%)	83 (46.9%)	
Age	59.4 (13.4)	59.8 (13.8)	59.1 (13.1)	0.624
Gender				0.053
Male	240 (67.8%)	129 (72.9%)	111 (62.7%)	
Female	114 (32.2%)	48 (27.1%)	66 (37.3%)	
M stage				0.250
M0	256 (98.8%)	121 (100%)	135 (97.8%)	
M1	3 (1.16%)	0 (0.00%)	3 (2.17%)	
N stage				0.622
N0	240 (98.4%)	121 (99.2%)	119 (97.5%)	
N1	4 (1.64%)	1 (0.82%)	3 (2.46%)	
Stage				0.014
I	166 (50.3%)	98 (58.3%)	68 (42.0%)	
II	80 (24.2%)	37 (22.0%)	43 (26.5%)	
III	80 (24.2%)	32 (19.0%)	48 (29.6%)	
IV	4 (1.21%)	1 (0.60%)	3 (1.85%)	
T stage				< 0.001
T1	176 (50.1%)	105 (60.0%)	71 (40.3%)	
T2	87 (24.8%)	37 (21.1%)	50 (28.4%)	
T3	75 (21.4%)	32 (18.3%)	43 (24.4%)	
T4	13 (3.70%)	1 (0.57%)	12 (6.82%)	
Treatment				0.456
Radiation therapy	186 (52.5%)	97 (54.8%)	89 (50.3%)	
Pharmaceutical therapy	168 (47.5%)	80 (45.2%)	88 (49.7%)	

### Risk score is remarkably associated with cell cycle

**Fig. 3:**
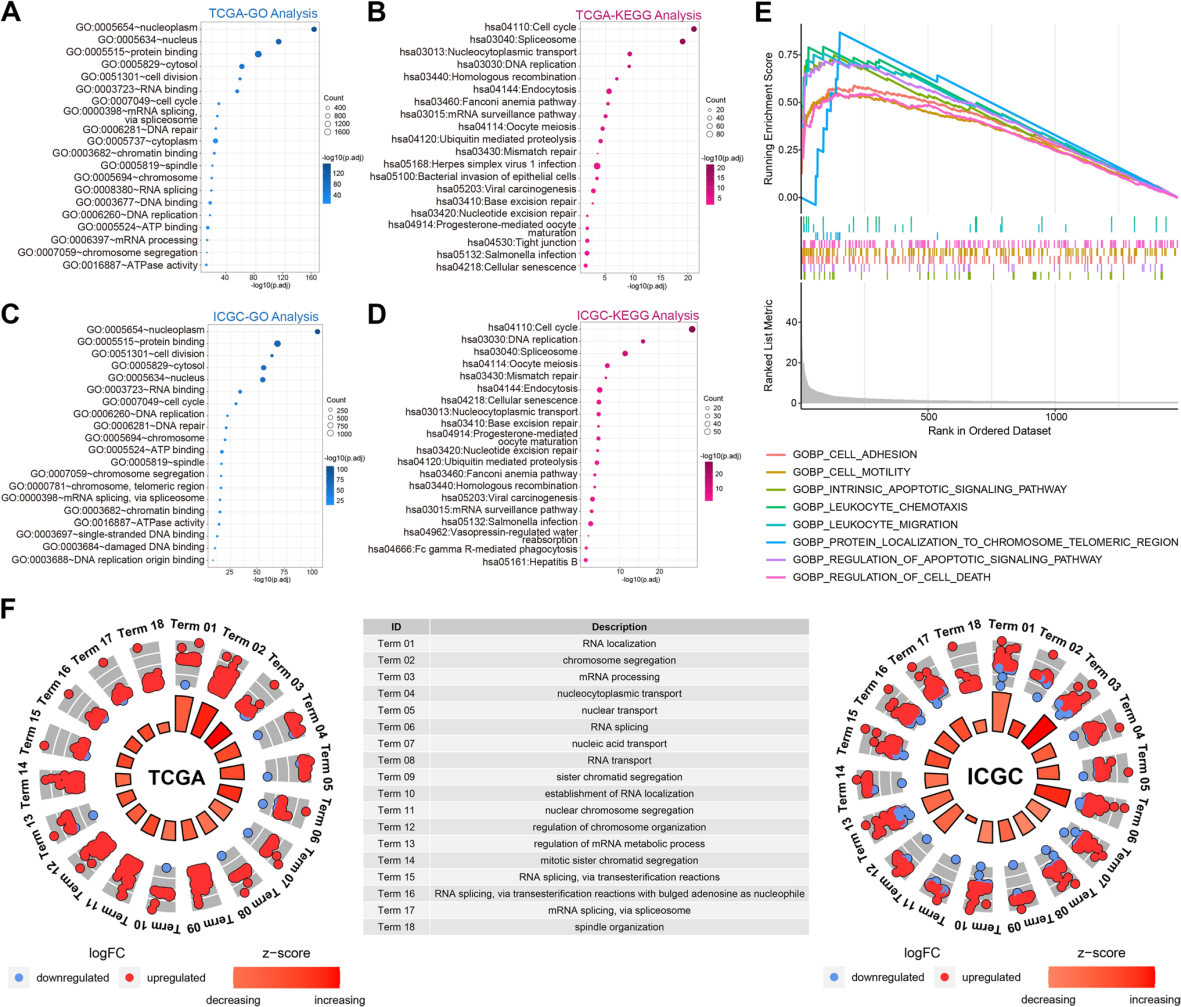
Biological functions associated with risk scores. **A–D** GO and KEGG analysis of risk score-related pathways in TCGA and ICGC. **E** GSEA analysis of genes upregulated in the high-risk group. **F** GO analysis of genes differentially expressed in the high-risk group

After filtrating genes most related to the risk score by conducting Spearman analysis (*p < *0.001 and *R* > 0.5), we obtained 2136 and 1354 genes in the training (Supplementary Material 2) and validation cohorts (Supplementary Material 3), respectively, to explore the biological functions and pathways. Through GO analysis, we found that the risk score was significantly associated with cell cycle-related biological functions, including cell division, DNA replication, nuclear division, and DNA repair, in both the TCGA and ICGC cohorts (Fig. 3A, C). KEGG analysis yielded similar results, with cell cycle, DNA replication, cellular senescence, and endocytosis being closely involved (Fig. 3B, D). Through GSEA and GO analysis, we analyzed the functional differences and identified several significant pathways in the enrichment of MSigDB collection (c5.go.v2022.1.Hs.symbols.gmt) in the high-risk group. These pathways included intrinsic apoptosis signaling pathway, cell adhesion, cell motility, leukocyte chemotaxis, and signaling receptor binding (Fig. 3E). GO analysis indicated that the significantly upregulated genes in the high-risk group were closely associated with cell cycle-related pathways, corroborating the results of the aforementioned correlation analysis (Fig. 3F). This result revealed that TRGs might be involved in uncontrolled cell cycles and cause worse prognosis in patients with HCC.

### Somatic mutation landscape between groups

**Fig. 4:**
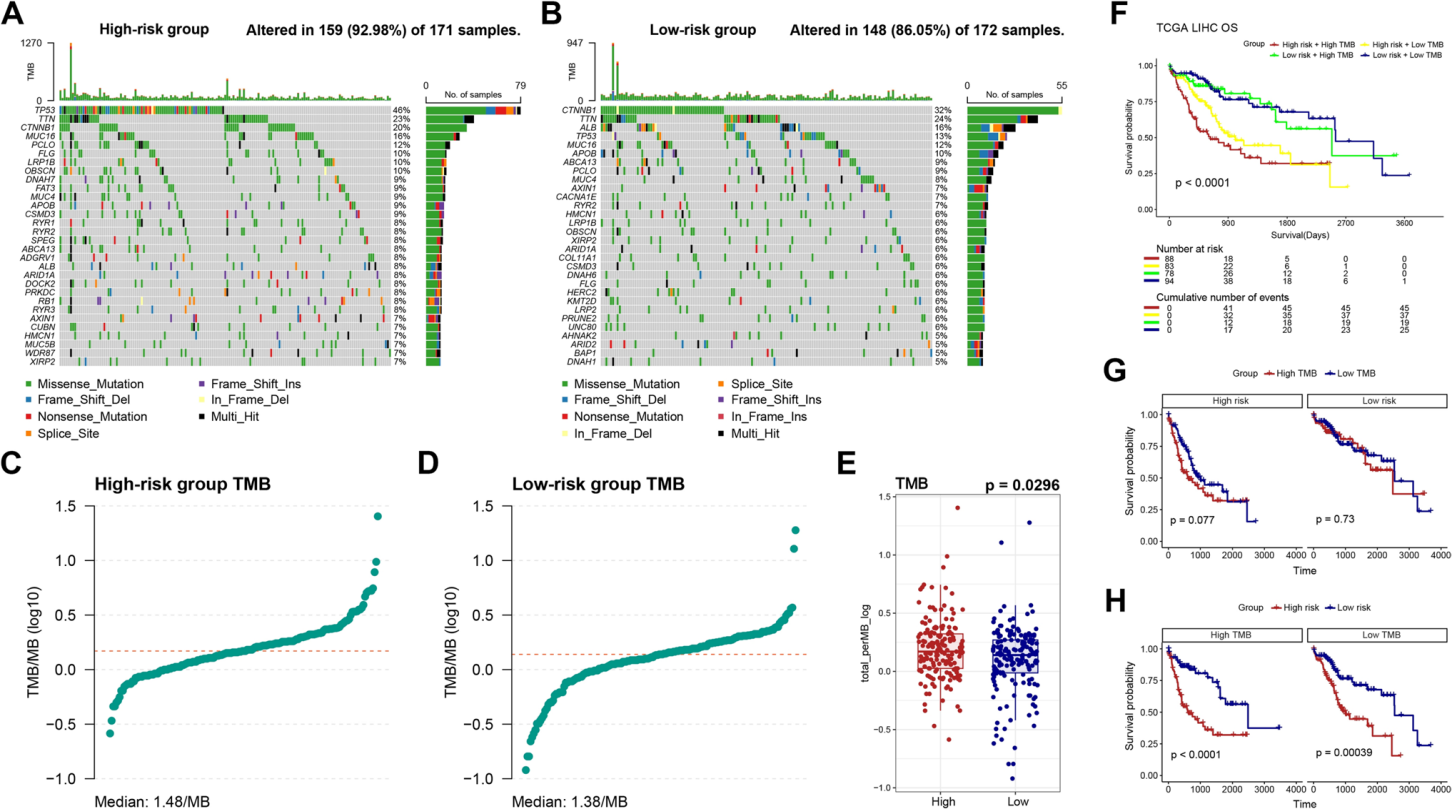
Mutation landscape of LIHC patients. **A**, **B** Comparison of the mutation landscape. **C–E** Comparison of TMB. **F–H** Survival analysis based on TMB and the risk score

As increased TMB can be caused by impaired cell cycle-related pathways; we compared the landscape of genetic mutation profiles between two groups (Fig. 4A, B). Notably, the mutation frequencies of the top 20 genes differed significantly between the groups. Higher TMB was observed in the high-risk group, which may be related to the high-risk score and poor prognosis (Fig. 4C–E). Moreover, on conducting a survival analysis based on TMB and risk score, no discernible differences were detected between patients with high TMB and those with low TMB in overall survival (Fig. S1). By conducting survival analysis combining TMB and risk scores, we found that patients with low-risk scores had the best prognosis regardless of their TMB levels, while patients exhibiting high TMB alongside high-risk scores tended to have the worst overall survival rate (Fig. 4F–H).

### Immune infiltration analysis

**Fig. 5:**
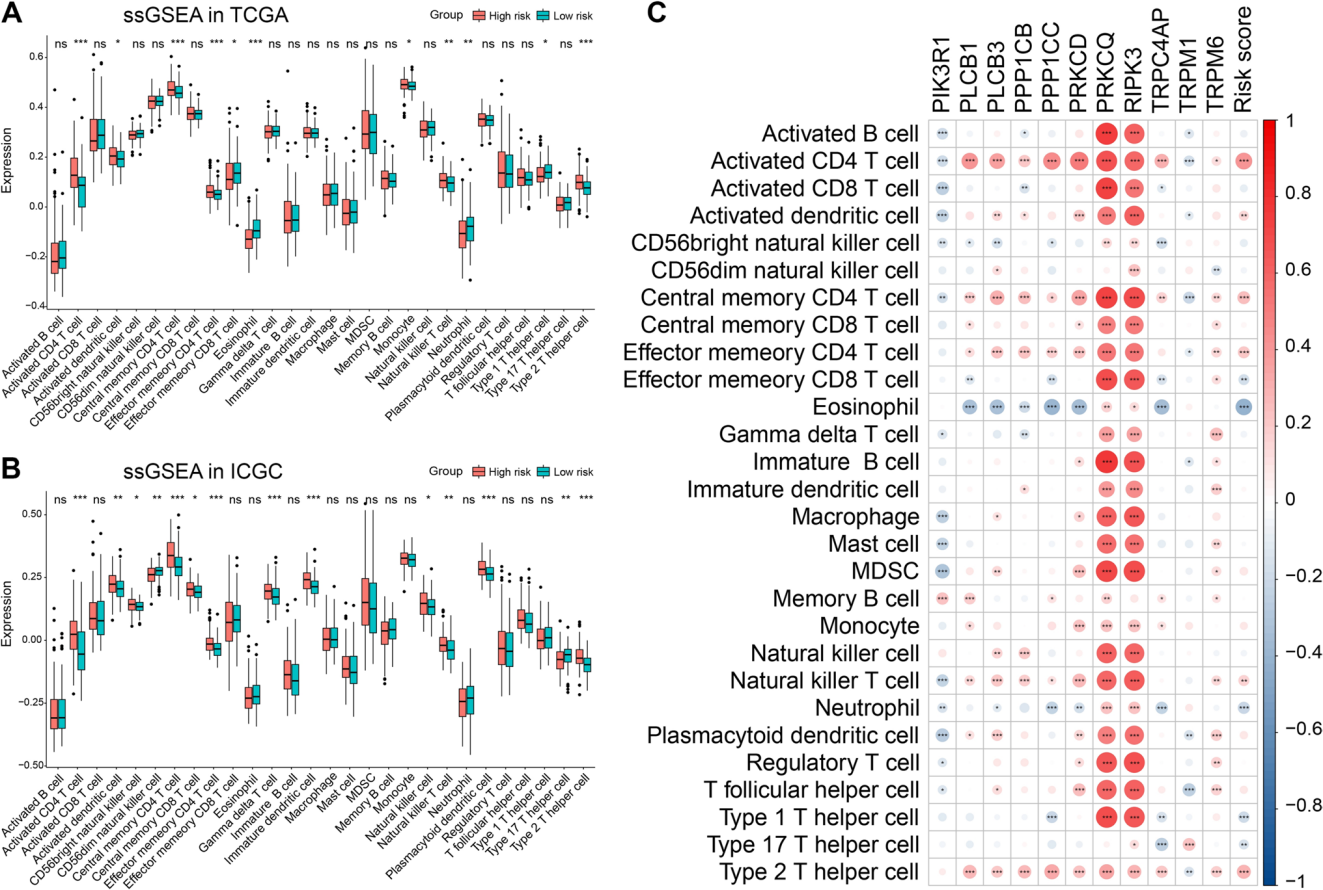
Immune infiltration analysis. **A** ssGSEA analysis in TCGA. **B** ssGSEA analysis in ICGC. **C** Correlation analysis between immune infiltrating cells and TRGs along with risk score. *p < 0.05, **p < 0.01, and ***p < 0.001. ns indicates *p* > 0.05

To examine the differences in immunological function, we conducted CIBERSORT and ssGSEA analyses. The CIBERSORT analysis revealed that the high-risk group exhibited a greater proportion of dendritic resting cells and M0 macrophages (Fig. S2). Additionally, ssGSEA analysis demonstrated that the expression of 11 immune cell subtypes, comprising activated CD4 T, activated dendritic, central memory CD4 T, central memory CD8 T, effector memory CD4 T, natural killer T, and type 2 T-helper cells, were considerably different, with most subtypes exhibiting high expression levels in the high-risk group (Fig. 5A). Similar results were observed in the validation cohort (Fig. 5B). The results of correlation analysis (Fig. 5C) suggested a more substantial infiltration of CD4 T-cells in the high-risk group.

### Comparison of the therapeutic effect

**Fig. 6:**
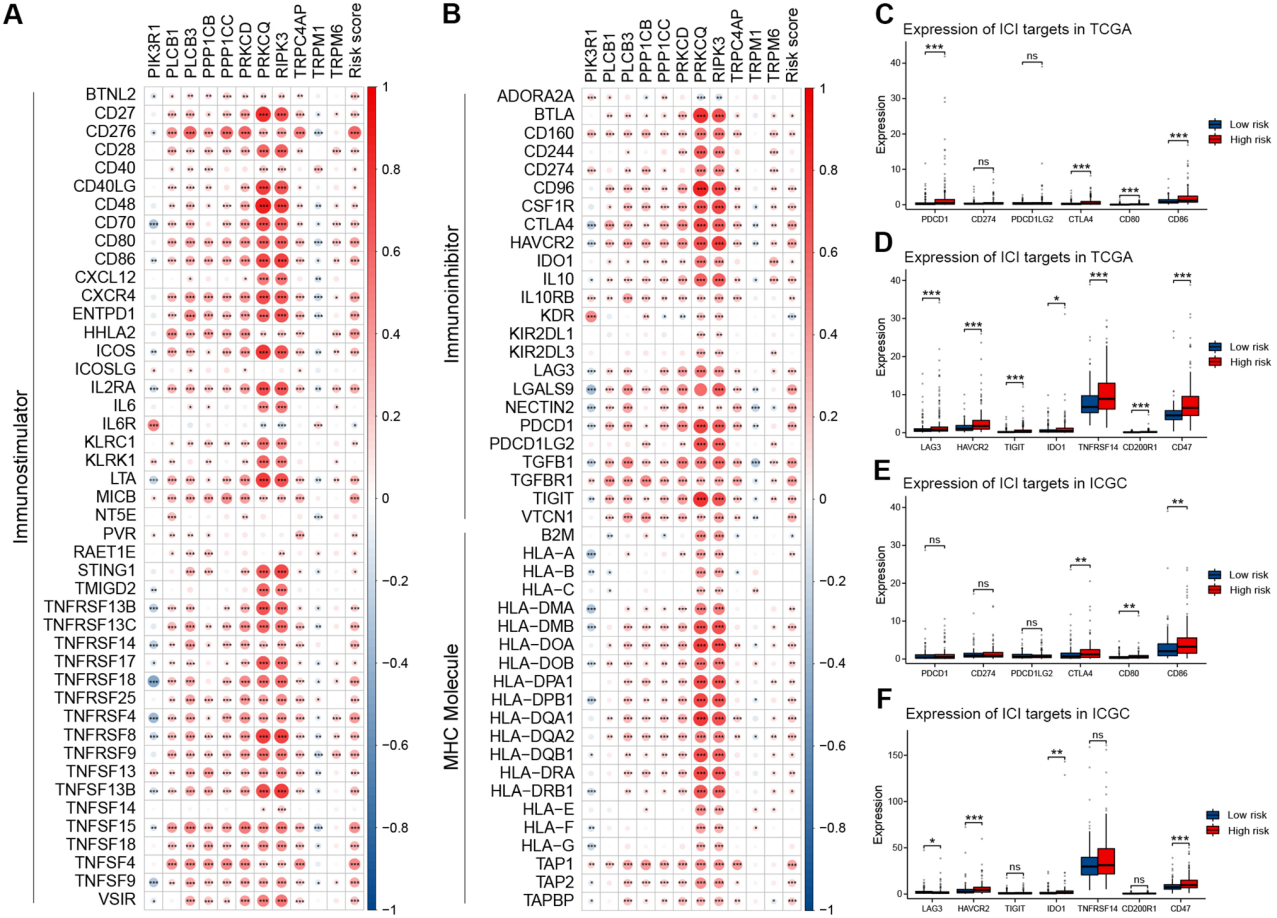
Comprehensive analysis of immunomodulator genes. **A**, **B** Correlation analysis between immunomodulatory genes, risk score, and TRGs. **C**, **D** Comparison of several immune checkpoint genes in TCGA. **E**, **F** Comparison of several immune checkpoint genes in ICGC. *p < 0.05, **p < 0.01, and ***p < 0.001. ns indicates *p* > 0.05

Immune checkpoint inhibitors (ICIs) play a pivotal role in HCC therapy. Correlation analysis between immune modulator genes, 11 TRGs, and risk scores (Fig. 6A, B) revealed a close correlation between the risk score and most immune modulator genes. Furthermore, we identified targets of immunomodulatory drugs under clinical trials for advanced HCC and compared the expression of these targets between the low-risk and high-risk groups. We discovered that most immunomodulatory targets (PDCD1, CTLA4, CD80, CD86, LAG3, HAVCR2, TIGHT, IDO1, TNFRSF14, and CD47) had notably higher expressions in the high-risk group (Fig. 6C–F).

**Fig. 7:**
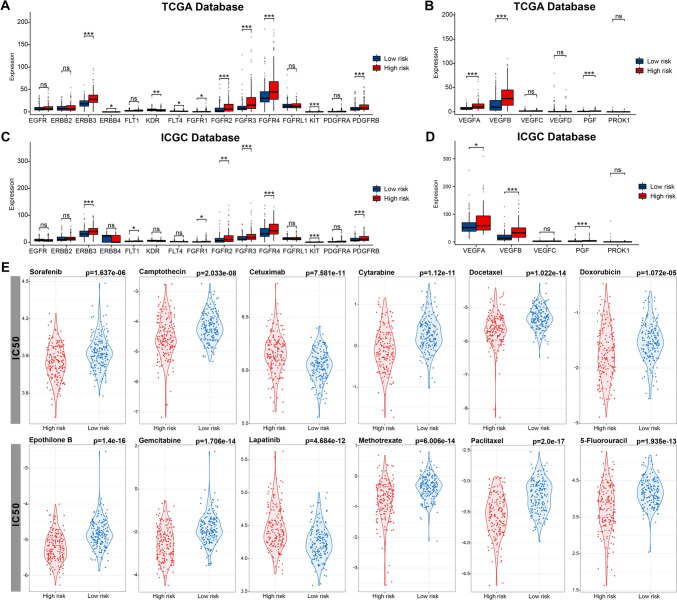
Evaluation of therapeutic effects. **A**, **B** Comparison of RTKs and VEGF family in TCGA. **C**, **D** Comparison of RTKs and VEGF family in ICGC. **E** Comparison of IC50 of different agents. *p < 0.05, **p < 0.01, and ***p < 0.001. ns indicates *p* > 0.05

Tyrosine kinase inhibitors (TKIs) and antiangiogenic drugs are pivotal in HCC treatment. We observed differences in the expression of various receptor tyrosine kinases (RTKs) and the VEGF family across the two risk groups. Several RTKs, including FGFR 1–4 and KIT, and VEGF families, comprising VEGFA, VEGFB, and PGF, exhibited substantial expressions in the high-risk group (Fig. 7A–D), while KDR (also known as VEGFR2) and FLT4 (also known as VEGFR3) displayed high expressions in the low-risk group.

Moreover, we identified 12 potential drugs by estimating IC50 values obtained from the GDSC database. The IC50 of most of these commonly used drugs, including fluorouracil, doxorubicin, gemcitabine, and paclitaxel, was lower in the high-risk group (Fig. 7E). The findings indicated that patients in the high-risk group would derive greater advantages from systemic therapy.

### Construction of nomogram for the prognostic model

**Fig. 8:**
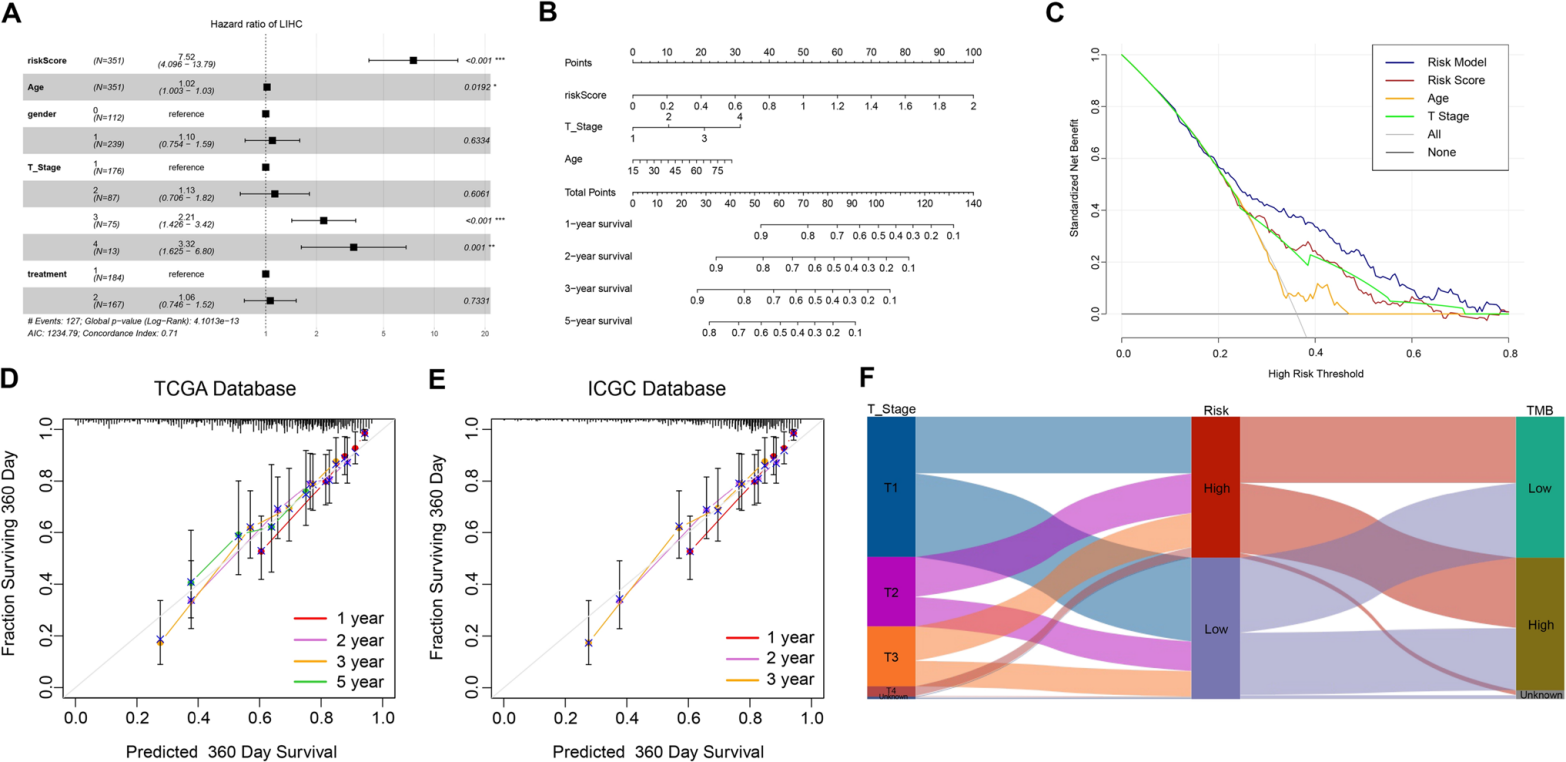
Establishment of the risk model. **A** Multivariate Cox regression analysis in TCGA. **B** Construction of individualized prediction model. **C** Evaluation of the risk model by DCA. **D**, **E** Evaluation of the risk model by a calibration curve. **F** Sankey diagram of LIHC patients

We conducted a multivariate Cox regression analysis on the risk score and clinicopathologic features to establish a risk model. Our findings revealed that independent risk factors included risk score, age, and T stage (Fig. 8A). Using these independent risk factors, we developed a prediction model and established a nomogram (Fig. 8B). Our risk model outperformed other approaches, such as in terms of age, T stage, and risk score, as confirmed by the decision curve analysis (Fig. 8C). The calibration curve showed a satisfactory match between the predicted survival events and the actual survival observations in both training and validation groups, indicating the accuracy of the prediction model (Fig. 8D, E). The Sankey diagram depicts the relationship between different T stages, risk scores, and TMB in patients with HCC (Fig. 8F).

### Transcription factor (TF)–TRG network analysis

**Fig. 9:**
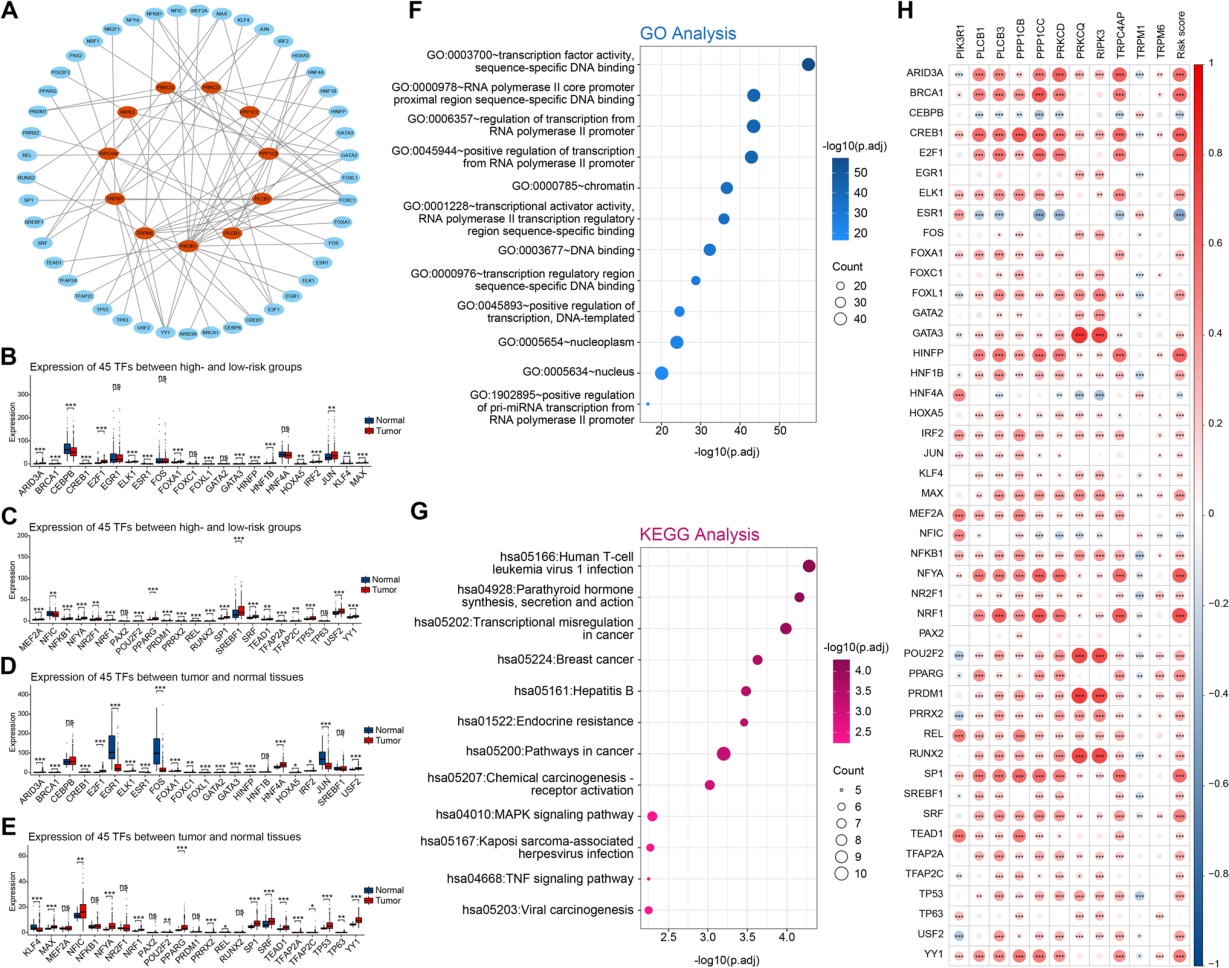
Identification of transcription factors. **A** Network of TFs and TRGs. **B**, **C** Comparison of TF expression. **D**, **E** Comparison of TF expression between tumor and normal tissues. **F**, **G** Biological functional analysis of TFs. **(H)** Correlation of TFs and TRGs along with risk score. *p < 0.05, **p < 0.01, and ***p < 0.001. *ns* indicates *p* > 0.05

Using the Human Protein Atlas (HPA), an online tool, we verified the expression of eight TRGs at the protein level by antibody staining, whereas three TRGs (*PRKCQ, RIPK3, and TRPM6*) lacked protein expression data (Fig. S3). Subsequently, we sought to identify the regulatory genes of TRGs. The development of HCC is aided by the dysregulation of liver-enriched TFs (Cai et al. 2017). Through NetworkAnalyst, an online tool providing comprehensive gene expression profiling and network visual analytics with the JASPAR TF binding site profile database-derived TF targets, we predicted 45 TFs and constructed a network with 11 TRGs (Fig. 9A). In our study, we observed upregulation of most TFs in both tumor tissues and the high-risk group (Figs. 9B–E). The results of biological functional analysis indicated that 45 TFs were significantly present in numerous cancer-related pathways such as chemical carcinogenesis, MAPK signaling pathway, transcriptional misregulation in cancer, and TNF signaling pathway (Figs. 9F, G). Correlation analysis demonstrated that TRGs and risk scores were closely associated with TFs (Fig. 9H), indicating that some genes in the 11 TRGs regulated by TFs might influence HCC progression through the aforementioned pathways.

## Discussion

Despite available curative and palliative treatments, the likelihood of tumor recurrence is high following HCC treatment, leading to a typically poor prognosis for patients (Llovet et al. 2008; Sherman 2008). The development of specialized therapy regimens requires the identification of prognostic indicators to improve the prognosis of HCC. TRPs are significant in the onset of HCC, indicating a prospective area for further research in hepatocellular carcinoma (Hu et al. 2018). However, no risk scoring system for HCC has been developed based on TRGs. Our research managed to establish 11 TRG-based risk model, providing new options for predicting and improving prognosis of patients with HCC.

We utilized LASSO Cox regression analysis to identify the optimal prognostic indicators and establish the risk scores for patients. The biological functional analysis indicated that risk score correlated with cell cycle, which when dysregulated may result in higher TMB. The TMB is a crucial factor that influences the overall survival of patients with HCC and their response to immunotherapy (Tang et al. 2021). Moreover, a higher TMB has been linked to the efficacy of immunotherapy, with increased TMB leading to better tumor remission effects and clinical benefits obtained from immunotherapy (Meléndez et al. 2018). Based on our results that showed higher TMB in the high-risk group, we postulate that individuals with high risk may gain significant benefits from immunotherapy.

Considering that individuals in the high-risk group are presumed to gain remarkable benefits from immunotherapy, we investigated whether differences existed in the immune microenvironment between the two groups. We concentrated on the differences in immune cell infiltration, which is closely linked to the response rate of immunotherapy in general. Although the unique immunological environment of the liver results in low immunotherapy response rates for HCC, substantial clinical evidence suggests that the immune cell composition of HCC tumors has a close association with the overall prognosis and response to therapy (Yasuoka et al. 2020). Our study found a strong association between CD4 T cell expression and risk score. According to reports, CD4 T cells inhibit cancer cell proliferation by hindering cell cycle progression at G1/S (Seung et al. 2022). The crucial genes of the cell cycle pathway that are strongly represented in the high-risk group may serve as potential participants in the anti-proliferation activity of CD4 T cells, when considered in conjunction with the outcomes of our functional enrichment analysis. This offers new insights and opportunities for conducting more comprehensive mechanistic studies.

Systemic therapy, referring mainly to anti-tumor therapy, including molecular-targeted drugs, immunotherapy, chemotherapy, and traditional Chinese medicine, plays a pivotal role in enhancing the prognosis of patients with HCC. The effectiveness of many treatments, however, differs among patients. For example, patients receiving ICIs are required to undergo genetic testing, whereas for patients taking TKIs and chemotherapeutic medications, there is no theoretical evidence of personalized dosing prior to administration. A detailed risk scoring system is urgently needed for personalized prediction of response rate to systemic therapy aiming to enhance treatment effectiveness and improve patient prognosis. Based upon our results, individualized treatment approach could be effectively developed using the risk scoring system, which predicts the efficacy of immunotherapy, molecular targeted therapy, and chemotherapy. In recent years, ICIs, TKIs, and VEGF inhibitors have emerged as promising options for cancer treatment (Callahan et al. 2016; European Association for the Study of the Liver. Electronic address: easloffice@easloffice.eu and European Association for the Study of the Liver 2018). The IMbrave150 trial established the combination of atezolizumab and bevacizumab as a first-line treatment option in patients (Finn et al. 2020). We conducted correlation analysis to investigate the relationship between risk score and immune checkpoint genes. Moreover, we compared the expression of immune checkpoint genes between the two groups. We found that the risk score significantly correlated with the expression of most immune checkpoint genes and their upregulation was generally observed in the high-risk group. Therefore, we believe that patients in the high-risk group would benefit more from combined immunotherapy and small-molecule targeted therapy. Notably, we found that all four members of the FGFR family, namely FGFR 1–4, were highly expressed in the high-risk group. This suggests that these patients may be more responsive to TKIs that target FGFR. In the field of chemotherapy, the FOLFOX4 regimen is approved in China as a first-line treatment for patients with locally advanced and metastatic HCC that cannot be treated with surgical resection or locoregional therapy (Qin et al. 2013). In a recent study, He et al. evaluated the combination of sorafenib and intraarterial FOLFOX (SoraHAIC) as a first-line treatment option for patients with HCC and portal vein thrombosis and observed positive results (He et al. 2019). We could predict the sensitivity to chemotherapy using the risk score. Analysis of the GDSC data revealed multiple drugs with lower IC50s in the high-risk group, suggesting a higher chemotherapy sensitivity in this group. Thus, patients in the high-risk group may have a higher response rate to treatments such as the FOLFOX4 regimen or SoraHAIC. The risk score might offer an adequate theoretical basis for choosing an optimal treatment strategy. Our study suggests that patients with high-risk scores may have a wider range of treatment options, while the lower response rates to systemic therapy observed in patients with low-risk scores may be due to TRGs and require further exploration.

The analysis of gene expression profiles has shown that liver-enriched TFs can downregulate the expression of most genes in HCC. This suggests that these TFs may function in suppressing the gene expression in HCC (Gong et al. 2020). Using online tools, we predicted 45 TFs that might be involved in HCC progression by regulating the expression of 11 TRGs. Given that many of these TFs were significantly upregulated or downregulated in the high-risk group, our study offers new insights into understanding the function of TFs in the progression of HCC, as determined by the risk score.

As far as we know, this is the first prognostic model for HCC that utilizes TRGs and has been validated using two independent databases TCGA and ICGC. We screened 11 TRGs, calculated their expression-based prognostic risk scores, and developed a risk model by integrating them with clinicopathologic factors. Using the risk scores, we evaluated the immune microenvironment and therapeutic effects in patients with HCC. Furthermore, we identified the corresponding sensitive drugs for each group based on the risk scores and constructed a nomogram that could accurately predict the overall survival. Despite its contributions, our study has some limitations. Some of the expression profiles in the ICGC dataset contained missing values, which were supplemented using the "impute.knn()" function. This may have introduced bias into our data. Besides, our study primarily focused on bioinformatic analysis without experimental validation. Further experimental analyses are required to explore the functions of the identified TRGs in HCC. This will help identify subgroups of HCC patients who would benefit from ICI or TKI therapy and to better understand the relationship between TRGs and HCC progression.

## Conclusion

In our study, we constructed a novel prognostic model using 11 TRGs for HCC. This model offers a new theoretical framework for predicting survival and developing individualized treatment strategies for patients with HCC. It is a clinically relevant contribution to the search for prognostic biomarkers and provides new insights into understanding the correlation between TRGs and HCC.

## Supplementary Information

Below is the link to the electronic supplementary material.

Supplementary file1 Figure S1 Survival Analysis of LIHC patients with high- and low-TMB. (PDF 8 KB)

Supplementary file2 Figure S2 CIBERSORT analysis of TCGA cohort. (A–B) CIBERSORT analysis of ICGC cohort. (C–D) (PDF 15307 KB)

Supplementary file3 Figure S3 Protein expression of TRGs from HPA. (PDF 40795 KB)

Supplementary file4 (ZIP 40 KB)

Supplementary file5 (DOCX 19 KB)

## Data Availability

The datasets generated during and/or analyzed during the current study are available from the corresponding author on reasonable request.

## Abbreviations

HCC: Hepatocellular carcinoma
TCGA: The Cancer Genome Atlas
GSEA: Gene set enrichment analysis
FDR: False discovery rate
GO: Gene ontology
KEGG: Kyoto Encyclopedia of Genes and Genomes
CIBERSORT: Cell-type identification by estimating relative subsets of RNA transcripts
ssGSEA: Single-sample gene set enrichment analysis
TRP: Transient receptor potential
TMB: Tumor mutation burden
ICI: Immune checkpoint inhibitor
RTK: Receptor protein tyrosine kinase
TKI: Protein receptor tyrosine kinase inhibitor
LASSO: Least absolute shrinkage and selection operator
ICGC: International Cancer Genome Consortium
