# Supplementary figures and images for: Identification of a TRP channel-related risk model for predicting prognosis and therapeutic effects of patients with hepatocellular carcinoma
Source: J Cancer Res Clin Oncol. 2023 Sep 21;149(18):16811–25. doi: 10.1007/s00432-023-05394-7 (PMC10645640; doi:10.1007/s00432-023-05394-7)

# TCGA LIHC OS

Group — High TMB — Low TMB

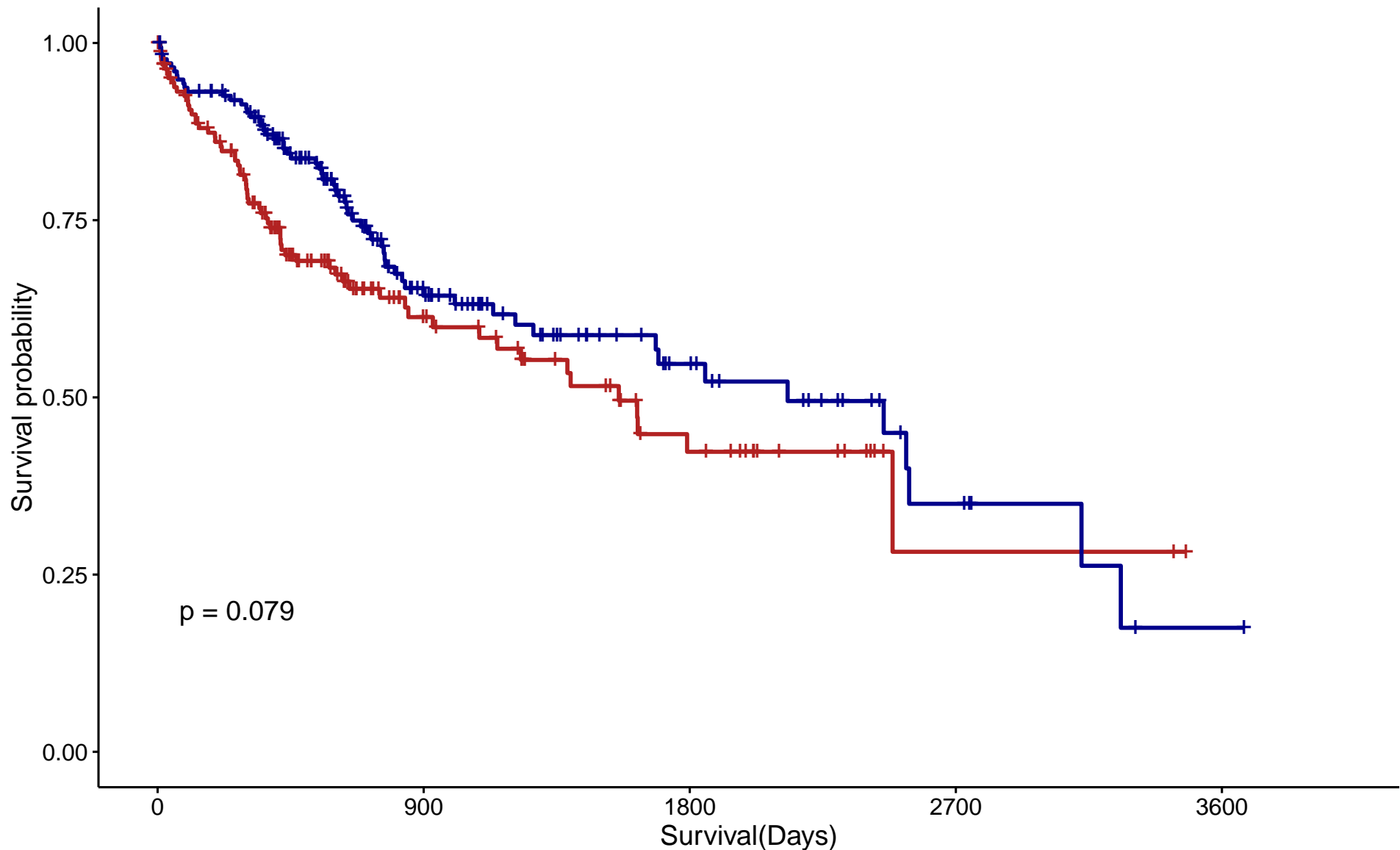

## Number at risk

|     |    |    |   |   |
|-----|----|----|---|---|
| 166 | 44 | 17 | 2 | 0 |
| 177 | 60 | 24 | 7 | 1 |

## Cumulative number of events

|   |    |    |    |    |
|---|----|----|----|----|
| 0 | 53 | 63 | 64 | 64 |
| 0 | 49 | 55 | 60 | 62 |

Supplement: Supplementary file 1 — Supplementary file1 Figure S1 Survival Analysis of LIHC patients with high- and low-TMB. (PDF 8 KB) [file 432_2023_5394_MOESM1_ESM.pdf]

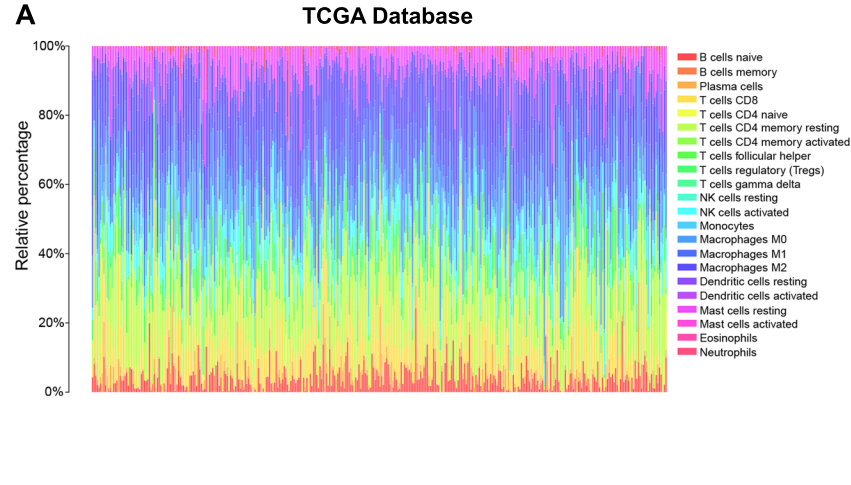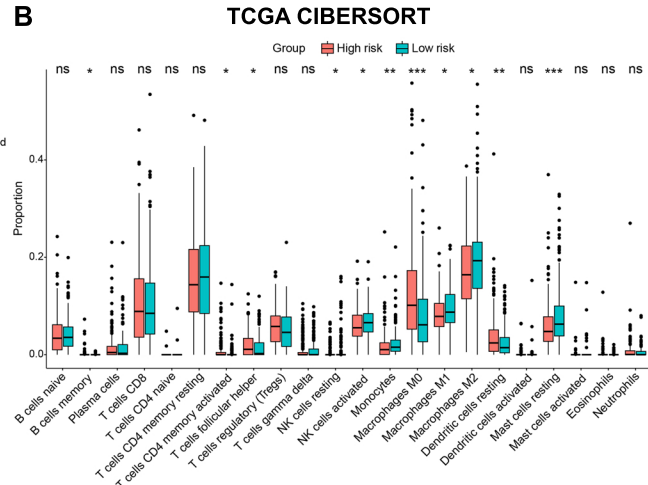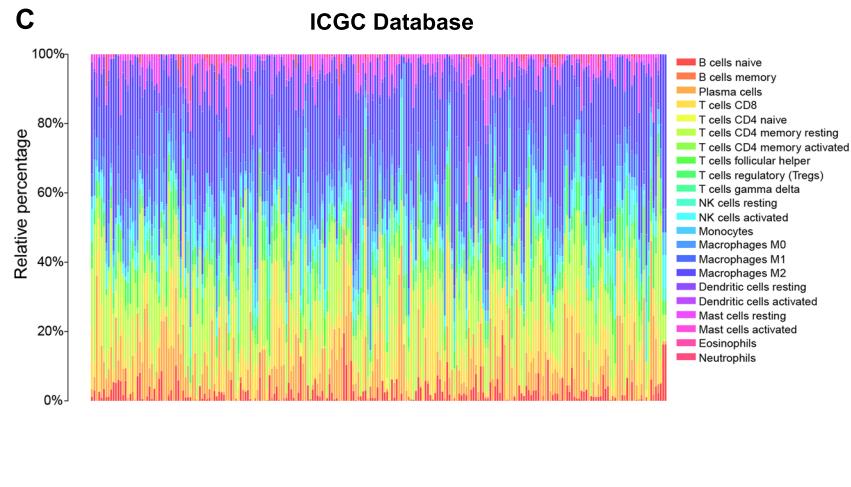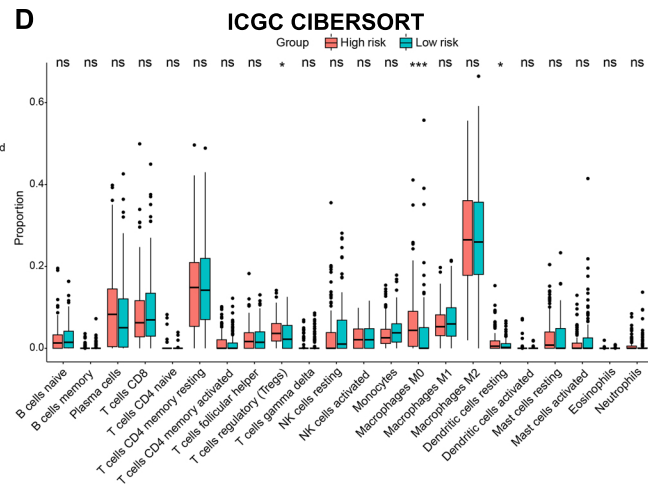

Supplement: Supplementary file 2 — Supplementary file2 Figure S2 CIBERSORT analysis of TCGA cohort. (A–B) CIBERSORT analysis of ICGC cohort. (C–D) (PDF 15307 KB) [file 432_2023_5394_MOESM2_ESM.pdf]

HCC

Normal

HCC

Normal

PIK3R1

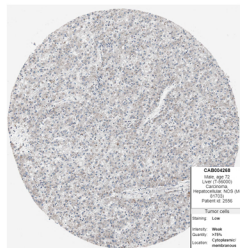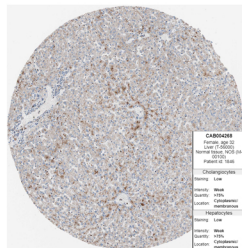

PPP1CC

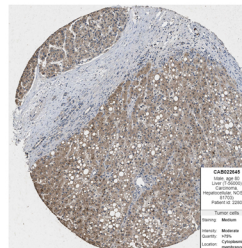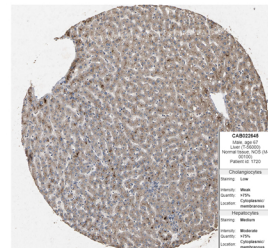

PLCB1

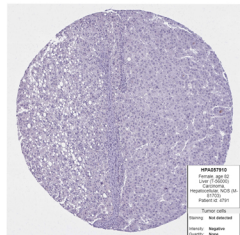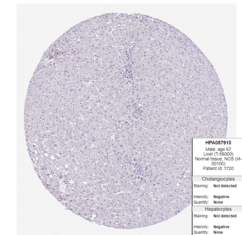

PRKCD

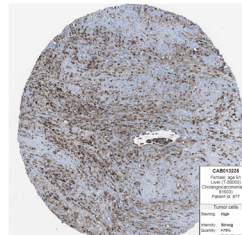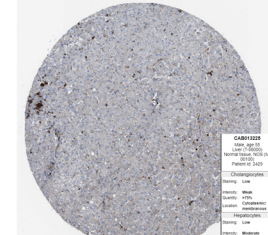

PLCB3

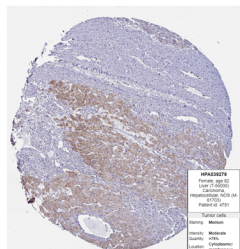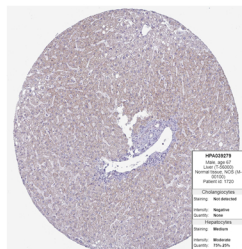

TRPC4AP

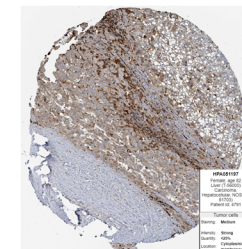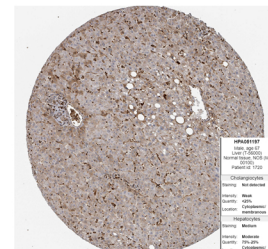

PPP1CB

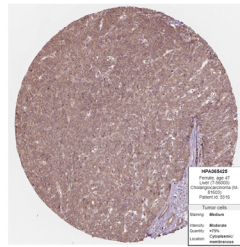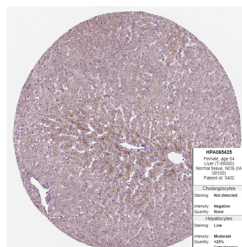

TRPM1

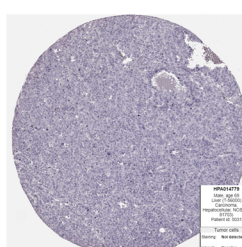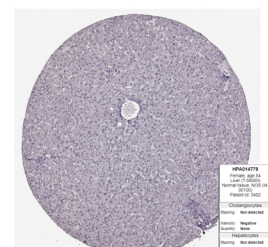

Supplement: Supplementary file 3 — Supplementary file3 Figure S3 Protein expression of TRGs from HPA. (PDF 40795 KB) [file 432_2023_5394_MOESM3_ESM.pdf]
