# Supplementary material for: Identification of a TRP channel-related risk model for predicting prognosis and therapeutic effects of patients with hepatocellular carcinoma
Source: J Cancer Res Clin Oncol. 2023 Sep 21;149(18):16811–25. doi: 10.1007/s00432-023-05394-7 (PMC10645640; doi:10.1007/s00432-023-05394-7)
Supplement: Supplementary file 5 — Supplementary file5 (DOCX 19 KB) [file 432_2023_5394_MOESM5_ESM.docx]

`Summary descriptives table of ICGC cohorts by groups of risk’

|  | **[ALL]** | **Low risk** | **High risk** | **p.overall** |
| --- | --- | --- | --- | --- |
|  | ***N=240*** | ***N=120*** | ***N=120*** |  |
| OS Time | 814 (419) | 888 (397) | 740 (430) | 0.006 |
| OS: |  |  |  | 0.007 |
| Alive | 197 (82.1%) | 107 (89.2%) | 90 (75.0%) |  |
| Dead | 43 (17.9%) | 13 (10.8%) | 30 (25.0%) |  |
| Gender: |  |  |  | 1.000 |
| Male | 179 (74.6%) | 90 (75.0%) | 89 (74.2%) |  |
| Female | 61 (25.4%) | 30 (25.0%) | 31 (25.8%) |  |
| Age | 67.5 (10.1) | 68.5 (9.45) | 66.5 (10.6) | 0.121 |
| T Stage: |  |  |  | 0.001 |
| T1 | 37 (15.4%) | 27 (22.5%) | 10 (8.33%) |  |
| T2 | 109 (45.4%) | 59 (49.2%) | 50 (41.7%) |  |
| T3 | 73 (30.4%) | 29 (24.2%) | 44 (36.7%) |  |
| T4 | 21 (8.75%) | 5 (4.17%) | 16 (13.3%) |  |
